# Development and validation of a predictive model for postoperative delirium in patients undergoing cardiac surgery

**DOI:** 10.3389/fcvm.2026.1758993

**Published:** 2026-06-04

**Authors:** Lin Cui, Jinhong Zhang, Xiaoling Sun, Jiangling Xia, Hongyu Xu

**Affiliations:** 1 School of Anesthesiology, Shandong Second Medical University, Weifang, Shandong, China; 2 Department of Anesthesiology, Zibo Central Hospital, Zibo, Shandong, China

**Keywords:** cardiac surgical procedures, delirium, logistic models, nomograms, risk factors

## Abstract

**Background:**

This study aimed to identify risk factors for postoperative delirium (POD) following adult cardiac surgery and to develop and validate a predictive nomogram for clinical application.

**Methods:**

Data from 724 cardiac surgery patients were randomly split into training (70%) and validation (30%) cohorts. Predictors selected through LASSO and multivariate logistic regression were used to build the nomogram. Model performance was examined using area under the receiver operating characteristic curve (AUROC), calibration plots, decision curve analysis (DCA), and clinical impact curve (CIC).

**Results:**

POD occurred in 285 patients (39.4%). Predictors included emergency surgery, age, Sequential Organ Failure Assessment score, postoperative shock, and postoperative blood lactate and glucose levels. The nomogram displayed excellent discriminative ability (high AUROC) and strong calibration agreement in both datasets. The model demonstrated strong clinical utility, as evidenced by the DCA and CIC results.

**Conclusion:**

This study identifies key risk factors for POD and presents a validated nomogram as an effective, clinically valuable tool to predict POD among patients undergoing cardiac surgery.

## 1 Introduction

Among adult patients undergoing cardiac surgery, postoperative delirium (POD) is a prevalent neuropsychiatric complication (1, 2). POD is characterized by acute fluctuations in consciousness and attention that typically emerge within the first few postoperative days (3, 4). The reported incidence of POD in cardiac surgery patients ranges from 11% to 55% (5). POD contributes to impairment of cognitive function and an elevated risk of physical injury. It is also associated with increased utilization of healthcare resources, imposing a substantial economic burden on both families and society (6). Given the substantial global burden of cardiovascular disease and the growing demand for cardiac surgical care, POD has increasingly become a critical focal point of clinical practice and research interest (7–9).

Despite its high prevalence, the precise pathophysiology of POD remains incompletely understood. Previous studies indicate that delirium onset is multifactorial and associated with a significant increase in patient mortality (10). Therefore, accurate risk stratification and timely preventive interventions are crucial for reducing the mortality rate (11). Additionally, evidence suggests that approximately 35% of delirium cases in the intensive care unit (ICU) are either underdiagnosed or misdiagnosed (12). These challenges highlight the critical need for efficient predictive models to assess POD risk in cardiac surgery patients. Notably, most existing prediction models primarily rely on preoperative risk factors (13, 14), often neglecting potentially influential intraoperative and postoperative variables. Therefore, there is a clear demand for a novel model that integrates data across the entire perioperative continuum (preoperative, intraoperative, and postoperative phases) to enable comprehensive risk assessment.

In the present study, we analyzed a comprehensive set of preoperative, intraoperative, and postoperative factors. Predictors of POD were selected through least absolute shrinkage and selection operator (LASSO) regression and multivariate logistic regression, the predictive nomogram was built using these predictors (15, 16). The performance of the model was assessed using discriminative ability, calibration, and clinical utility. The objective of this study was to provide a validated, clinically practical tool for predicting the risk of POD following cardiac surgery.

## 2 Methods

### 2.1 Study design

The study was on the basis of a retrospective cohort from a single center, and its reporting followed the TRIPOD (Transparent Reporting of a multivariable prediction model for Individual Prognosis or Diagnosis) statement. The model was intended to be applied from hospital admission to 24 h postoperatively. By collecting risk factors within this time window, the prediction model was constructed to estimate the potential of postoperative delirium.

### 2.2 Study population

Patients undergoing cardiac surgery at the Department of Cardiac and Great Vascular Surgery, Zibo Central Hospital, Shandong, China, between May 22, 2023, and March 31, 2025 were included in this retrospective analysis. This investigation was approved by the Ethics Review Committee of Zibo Central Hospital (Registration number: ChiCTR2300071454). The ethics committee waived the requirement for informed consent. Data collection was completed independently by two investigators, with one investigator entering the data into the system and the other investigator verifying its accuracy. The criteria for inclusion were: (1) patients aged ≥18 years and (2) patients undergoing open cardiovascular surgery, specifically cardiac valve replacement (CVR), coronary artery bypass grafting (CABG), combined CABG and CVR, or excision of a pericardial tumor. The criteria for exclusion were: (1) age < 18 years; (2) pre-existing cognitive impairment; (3) cardiac interventional procedures, including coronary angiography, coronary stent implantation, percutaneous balloon mitral valvuloplasty, or transcatheter aortic valve replacement; (4) postoperative mortality occurring within 1 week; and (5) missing or incomplete clinical data.

### 2.3 Candidate predictors

A comprehensive set of candidate predictors for postoperative delirium in patients undergoing cardiac surgery was selected based on prior literature, clinical expertise, and data availability (17, 18). The candidate predictors were categorized as preoperative, intraoperative, and postoperative factors. Preoperative predictors included age, sex, BMI, education, diabetes, NEUT, hemoglobin (Hgb), platelet count (PLT), and troponin I (TnI). Intraoperative predictors comprised surgical type, emergency surgery, cardiopulmonary bypass, remimazolam, dexmedetomidine, and intraoperative blood salvage. Postoperative predictors included postoperative lactate (post-op Lac), postoperative glucose (post-op Glu), postoperative C-reactive protein (post-op CRP), postoperative troponin I (post-op TnI), duration of surgery, SOFA score, postoperative shock, and postoperative reoperation.

### 2.4 Shock assessment

Starting from ICU admission, patients were assessed for shock three times per day during the first 24 h, primarily to determine whether shock occurred (yes/no). Shock was defined according to the 2022 Society for Cardiovascular Angiography and Interventions (SCAI) Shock Stages Classification.

### 2.5 Outcome

Delirium was the primary outcome of this study. All patients were transferred to the ICU immediately after surgery. Delirium assessments were initiated immediately after surgery and performed daily for 7 consecutive postoperative days by two trained researchers. Additional evaluations were conducted on observing or suspecting a change in mental status. Initial assessment of sedation levels was performed using the Richmond Agitation Sedation Scale (RASS). The score below −3 were considered deeply sedated and ineligible for delirium screening. For patients with a RASS score ≥ −3, delirium was assessed using the Confusion Assessment Method for the ICU (CAM-ICU) in accordance with established diagnostic criteria (19, 20).

### 2.6 Sample size

Previous methodological guidance suggests that logistic regression typically requires at least 10 outcome events per variable (21, 22). After variable selection using LASSO and multivariable logistic regression, six predictors were retained in the final model. In the training cohort, the incidence of postoperative delirium was 38.1%; therefore, the minimum number of delirium events required was 10 × 6 = 60, corresponding to an estimated minimum sample size of 10 × 6/0.381 ≈ 158. In our training dataset, 507 patients were included, of whom 193 developed delirium, which substantially exceeds the minimum requirement for model development, supporting the adequacy of the sample size for model development and internal validation.

### 2.7 Missing data

In this study, there were no missing data for the variables included in the model development dataset. Therefore, no imputation or case exclusion due to missingness was required. In clinical practice, if some predictors are unavailable at the time of use, real-time imputation can be applied to impute missing values prior to risk calculation.

### 2.8 Predictors selection and model development

Free Statistics Software (version 2.1.1) was used for data processing and analysis. A 7:3 random split was used to allocate patients into training and validation sets. The univariate regression analysis and the LASSO method were first applied to identify significant predictors of POD. The variables retained were then entered into a multivariable logistic regression model in the training set to develop the prediction model. Multicollinearity among predictors was assessed by the variance inflation factor (VIF), with a VIF of ≥5 considered indicative of collinearity. Based on the final model, a nomogram was then constructed to visually estimate the probability of POD.

### 2.9 Model validation

The validation cohort was used to evaluate the predictive performance. The C-index was calculated to quantify the discriminative performance of nomogram, reflecting its capacity to distinguish between high- and low-risk individuals. The predictive performance was evaluated using AUROC. The calibration curve was used to assess whether the probabilities predicted by the nomogram were consistent with the actual observed event rates. Bootstrapping validation (500 bootstrap resamples) was used to determine the calibration performance of the model and to reduce overfitting-induced bias.

### 2.10 Performance assessment

Clinical utility was assessed by applying decision curve analysis (DCA) and the clinical impact curve (CIC) to the nomogram.

### 2.11 Data analysis

Continuous variables following a normal distribution were presented as the mean ± SD, and group comparisons were conducted with Student’ s t-test. Median (interquartile range) was used to describe non-normally distributed variables, which were analyzed via the Mann–Whitney U test. Categorical data are reported as frequencies and percentages, and comparisons between groups were conducted with the *χ*^2^ test or Fisher’ s exact test. Statistical significance was defined as a two-tailed *p* value < 0.05.

## 3 Results

### 3.1 Characteristics of the study population

**Figure 1:**
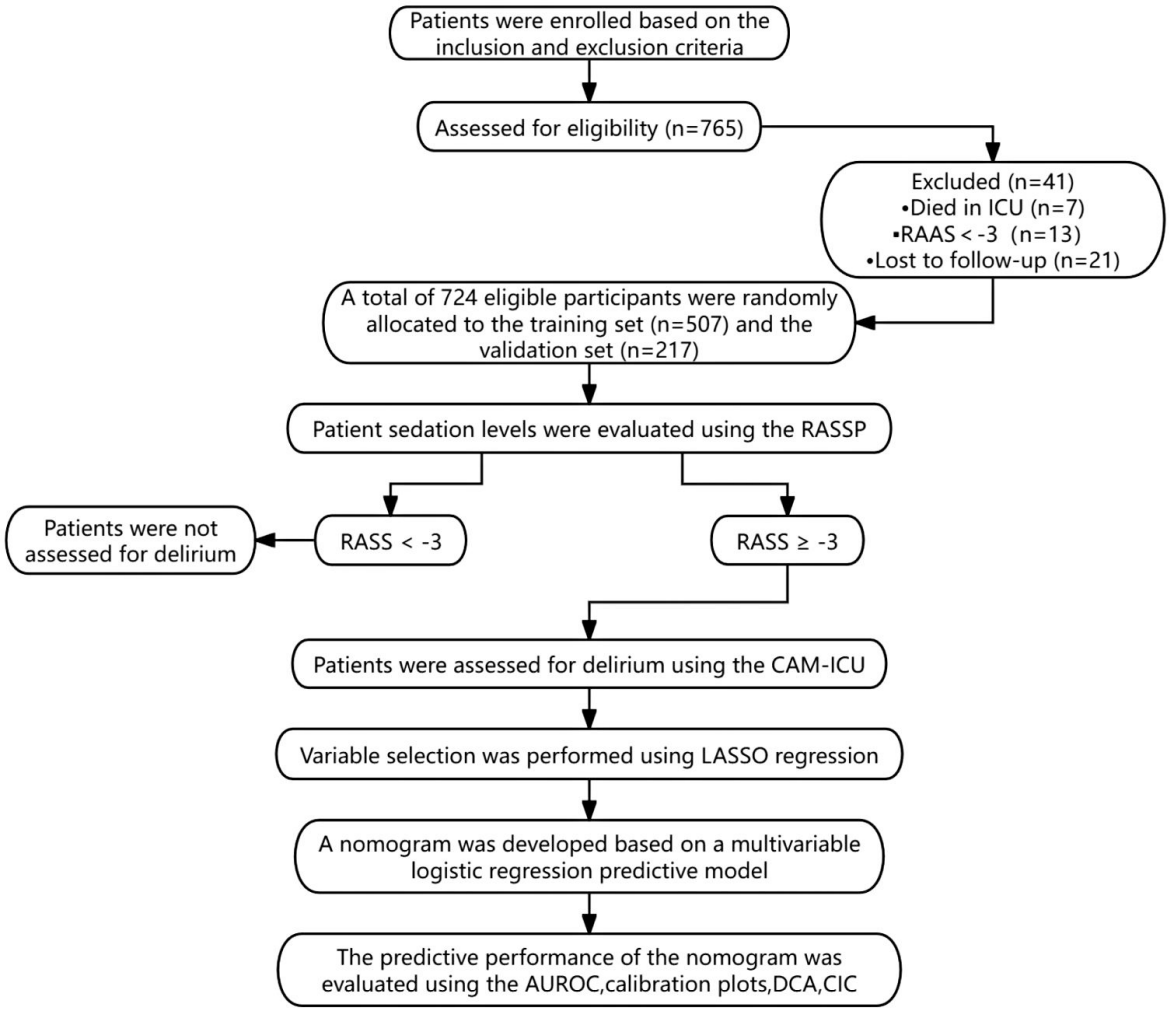
Study flowchart illustrating the construction and validation of the prediction model for postoperative delirium.

The study flow diagram is shown in Figure 1. A total of 765 patients were initially included. During the study, 41 patients were excluded: 7 died in the ICU, 13 had a RASS score < −3, and 21 were lost to follow-up. In total, 724 cardiac surgery patients were enrolled, with 507 (70%) assigned to the training set and 217 (30%) to the validation set. Baseline characteristics did not show significant difference between the groups, supporting the effectiveness of the randomization process. The overall cohort included 528 males (72.9%) and 196 females (27.1%), with a mean age of 64.5 ± 8.7 years. POD occurred in 285 patients (39.4%), including 193 and 92 patients in the training and validation sets, respectively. A total of 23 variables encompassing preoperative, intraoperative, and postoperative factors were included in the analysis (Table 1).

**Table 1:** The baseline characteristics of patients in training and validation sets.

Variables	Total	Training set	Validation set	*P-value*
	(*n* = 724)	(*n* = 507)	(*n* = 217)	
Delirium, *n* (%)				0.275
Non-Delirium	439 (60.6)	314 (61.9)	125 (57.6)	
Delirium	285 (39.4)	193 (38.1)	92 (42.4)	
Preoperative variables				
Age, year, Mean ± SD	64.5 ± 8.7	64.5 ± 8.6	64.4 ± 9.0	0.829
Gender, *n* (%)				0.892
Male	528 (72.9)	369 (72.8)	159 (73.3)	
Female	196 (27.1)	138 (27.2)	58 (26.7)	
BMI, *n* (%)				0.584
Underweight	11 (1.5)	7 (1.4)	4 (1.8)	
Normal weight	244 (33.7)	176 (34.7)	68 (31.3)	
Overweight	469 (64.8)	324 (63.9)	145 (66.8)	
Education, *n* (%)				0.725
Illiteracy	33 (4.6)	24 (4.7)	9 (4.1)	
Primary-School	224 (30.9)	163 (32.1)	61 (28.1)	
Middle-School	274 (37.8)	188 (37.1)	86 (39.6)	
High-School	135 (18.6)	90 (17.8)	45 (20.7)	
University	58 (8.0)	42 (8.3)	16 (7.4)	
Diabetes, *n* (%)				0.502
No	447 (61.7)	309 (60.9)	138 (63.6)	
Yes	277 (38.3)	198 (39.1)	79 (36.4)	
NEUT, 10^9^/L, Mean ± SD	4.3 ± 2.3	4.3 ± 2.4	4.3 ± 2.2	0.790
Hgb, g/L, Mean ± SD	134.3 ± 16.8	134.8 ± 16.9	133.1 ± 16.7	0.213
PLT, 10^9^/L, Mean ± SD	213.5 ± 62.5	213.7 ± 64.2	213.2 ± 58.6	0.923
TnI, ng/mL, Median (IQR)	0.0 (0.0, 0.1)	0.0 (0.0, 0.1)	0.0 (0.0, 0.1)	0.415
Intraoperative variables				
Type, *n* (%)				0.257
OPCAB	479 (66.2)	341 (67.3)	138 (63.6)	
CVR	63 (8.7)	48 (9.5)	15 (6.9)	
CABG + CVR	109 (15.1)	70 (13.8)	39 (18)	
AD	26 (3.6)	18 (3.6)	8 (3.7)	
ONCAB	11 (1.5)	5 (1)	6 (2.8)	
Others	36 (5.0)	25 (4.9)	11 (5.1)	
Emergency, *n* (%)				0.414
No	694 (95.9)	488 (96.3)	206 (94.9)	
Yes	30 (4.1)	19 (3.7)	11 (5.1)	
Cardiopulmonary Bypass, *n* (%)				0.347
No	482 (66.6)	343 (67.7)	139 (64.1)	
Yes	242 (33.4)	164 (32.3)	78 (35.9)	
Remimazolam, *n* (%)				0.486
Yes	86 (11.9)	63 (12.4)	23 (10.6)	
No	638 (88.1)	444 (87.6)	194 (89.4)	
Dexmedetomidine, *n* (%)				0.254
Yes	85 (11.7)	55 (10.8)	30 (13.8)	
No	639 (88.3)	452 (89.2)	187 (86.2)	
Intraoperative Blood Salvage, *n* (%)				0.457
No	122 (16.9)	82 (16.2)	40 (18.4)	
Yes	602 (83.1)	425 (83.8)	177 (81.6)	
Postoperative variables				
post-op Lac, mmol/L, Median (IQR)	1.5 (1.1, 2.3)	1.5 (1.1, 2.3)	1.4 (1.1, 2.3)	0.520
post-op Glu, mmol/L, Mean ± SD	8.9 ± 2.3	9.0 ± 2.4	8.7 ± 2.2	0.106
post-op CRP, mg/L, Mean ± SD	62.0 ± 30.7	62.2 ± 30.0	61.4 ± 32.4	0.740
post-op TnI, ng/mL, Median (IQR)	1.6 (0.6, 5.2)	1.6 (0.6, 5.6)	1.6 (0.6, 4.7)	0.942
Duration of surgery, minute, Mean ± SD	358.5 ± 92.5	357.9 ± 90.6	359.9 ± 97.1	0.787
SOFA score, *n* (%)				0.592
SOFA < 5	443 (61.2)	307 (60.6)	136 (62.7)	
SOFA≥5	281 (38.8)	200 (39.4)	81 (37.3)	
post-op Shock, *n* (%)				0.555
No	636 (87.8)	443 (87.4)	193 (88.9)	
Yes	88 (12.2)	64 (12.6)	24 (11.1)	
post-op Reoperation, *n* (%)				1.000
No	712 (98.3)	498 (98.2)	214 (98.6)	
Yes	12 (1.7)	9 (1.8)	3 (1.4)	

BMI, body mass index; NEUT, neutrophil; Hgb, hemoglobin; PLT, platelet; TnI, troponin I; OPCAB, off-pump coronary artery bypass; CVR, cardiac valve replacement; AD, aortic dissection; ONCAB, on-pump coronary artery bypass; Lac, lactate; Glu, Glucose; post-op, postoperative; CRP, C-reactive protein.

### 3.2 Identification of predictors of POD

**Figure 2:**
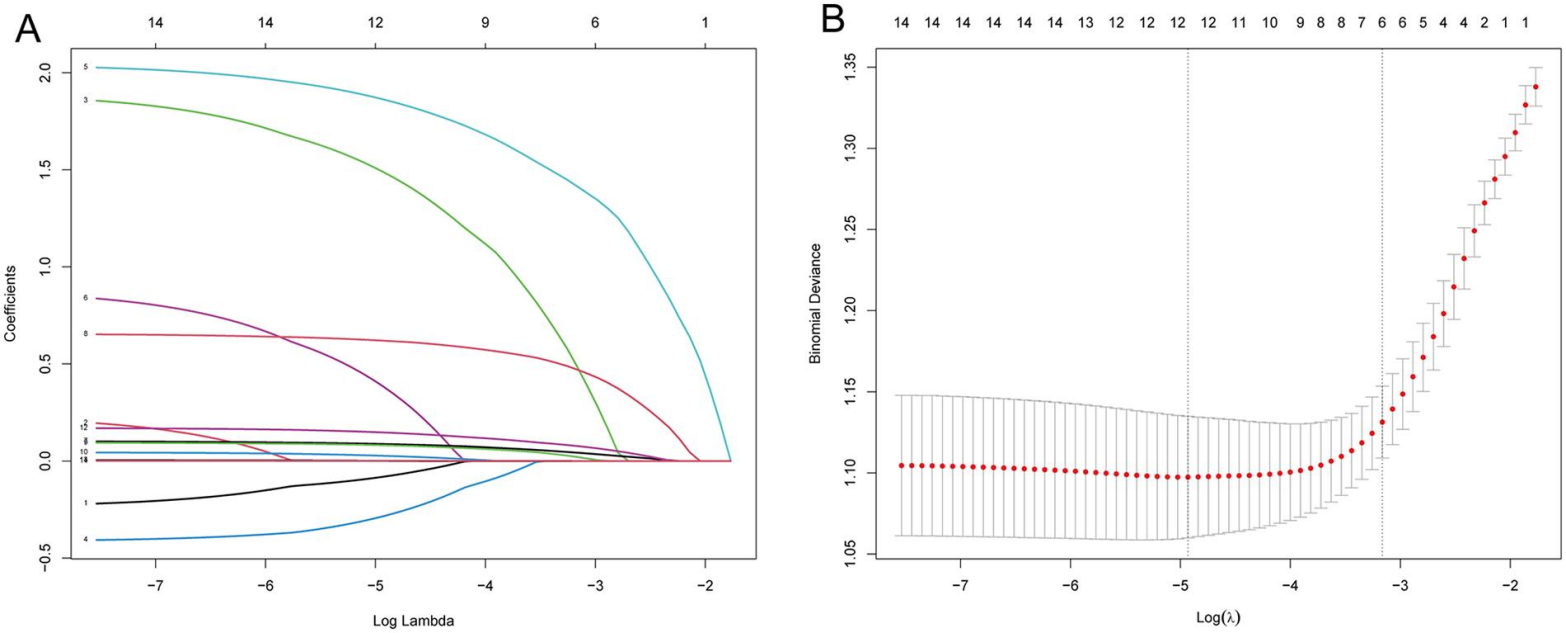
The predictors of the nomogram were screened using LASSO regression analysis in the training set. **(A)** Profiles of the coefficients for candidate variables across log(*λ*) values. **(B)** Ten-fold cross-validation was used to determine the optimal *λ*. The dotted vertical lines represent the *λ* values selected according to the minimum criterion and the 1-standard-error criterion.

First, we performed univariable logistic regression to screen predictors associated with postoperative delirium among the 23 candidate variables. The results showed that 14 clinical characteristics were significantly associated with postoperative delirium, including age, diabetes, NEUT, PLT, type of surgery, emergency, cardiopulmonary bypass, post-op Lac, post-op Glu, post-op TnI, duration of surgery, SOFA score, post-op shock, post-op reoperation (Table 2). Because the number of candidate variables was relatively large in relation to the number of outcome events, LASSO regression analysis enabled variable selection and coefficient shrinkage, thereby reducing the risk of overfitting. Therefore, to minimize overfitting and enhance model robustness, these 14 candidate variables were screened by LASSO regression analysis (Figure 2). This approach identified six potential predictors: emergency surgery, postoperative shock (post-op Shock), age, Sequential Organ Failure Assessment (SOFA) score, postoperative lactate (post-op Lac) level, and postoperative glucose (post-op Glu) level. A multivariate logistic regression analysis was then performed using these variables (Table 3). The results confirmed that all six factors remained statistically significant (*p* < 0.05), establishing them as independent predictors of POD. We also assessed multicollinearity among the six predictors using VIF. All VIF values were < 5, indicating no significant multicollinearity.

**Table 2:** The univariate logistic regression analysis of the predictors.

Variable	OR(0.95CI)	*P*_value
Preoperative variables		
Age, year	1.05 (1.03∼1.07)	<0.001
Gender		
Male		
Female	1.09 (0.78∼1.52)	0.626
BMI		
Underweight		
Normal weight	0.65 (0.19∼2.2)	0.492
Overweight	0.85 (0.25∼2.81)	0.786
Education		
Illiteracy		
Primary-School	0.9 (0.44∼1.88)	0.788
Middle-School	0.55 (0.27∼1.15)	0.112
High-School	0.69 (0.32∼1.48)	0.336
University	0.52 (0.22∼1.24)	0.14
Diabetes		
No		
Yes	0.71 (0.52∼0.96)	0.028
NEUT, 10^9^/L	1.13 (1.05∼1.2)	0.001
Hgb, g/L	1 (0.99∼1)	0.309
PLT, 10^9^/L	1 (0.99∼1)	0.001
TnI, ng/mL	1.07 (1∼1.15)	0.067
Intraoperative variables		
Type		
OPCAB		
CVR	1.38 (0.81∼2.34)	0.237
CABG + CVR	1.26 (0.82∼1.92)	0.289
AD	9.47 (3.21∼27.92)	<0.001
ONCAB	2.07 (0.62∼6.87)	0.236
Others	0.42 (0.18∼0.97)	0.042
Emergency		
No		
Yes	10.92 (3.77∼31.63)	<0.001
Cardiopulmonary Bypass		
No		
Yes	1.42 (1.04∼1.95)	0.027
Remomazolam		
No		
Yes	1.17 (0.74∼1.87)	0.503
Dexmedetomidine		
No		
Yes	0.74 (0.47∼1.16)	0.192
Intraoperative Blood Salvage		
No		
Yes	1.04 (0.7∼1.56)	0.835
Postoperative variables		
post-op Lac, mmol/L	1.33 (1.19∼1.49)	<0.001
post-op Glu, mmol/L	1.24 (1.16∼1.33)	<0.001
post-op CRP, mg/L	1 (0.99∼1)	0.352
post-op TnI, ng/mL	1.04 (1.02∼1.06)	<0.001
Duration of surgery, minute	1 (1∼1.01)	<0.001
SOFA score		
SOFA < 5		
SOFA≥5	3.36 (2.46∼4.6)	<0.001
post-op Shock		
No		
Yes	11.7 (6.35∼21.57)	<0.001
post-op Reoperation		
No		
Yes	4.74 (1.27∼17.66)	0.02

BMI, body mass index; NEUT, neutrophil; Hgb, hemoglobin; PLT, platelet; TnI, troponin I; OPCAB, off-pump coronary artery bypass; CVR, cardiac valve replacement; AD, aortic dissection; ONCAB, on-pump coronary artery bypass; Lac, lactate; Glu, Glucose; post-op, postoperative; CRP, C-reactive protein.

**Table 3:** The multivariate logistic regression analysis and multicollinearity analysis of the predictors.

Variable	OR (95%CI)	VIF	*p* value
Age, year	1.1 (1.08∼1.13)	1.193	<0.001
Emergency surgery		1.124	
No			
Yes	8.12 (2.23∼29.53)		0.001
post-op Shock		1.188	
No			
Yes	7.85 (3.76∼16.39)		<0.001
SOFA		1.091	
SOFA < 5			
SOFA≥5	2.08 (1.44∼3)		<0.001
post-op Lac (mmol/L)	1.14 (1.01∼1.29)	1.114	0.04
post-op Glu (mmol/L)	1.17 (1.08∼1.26)	1.030	<0.001

Lac, lactate; Glu, Glucose; post-op, postoperative.

### 3.3 Establishment and validation of a nomogram to predict POD

**Figure 3:**
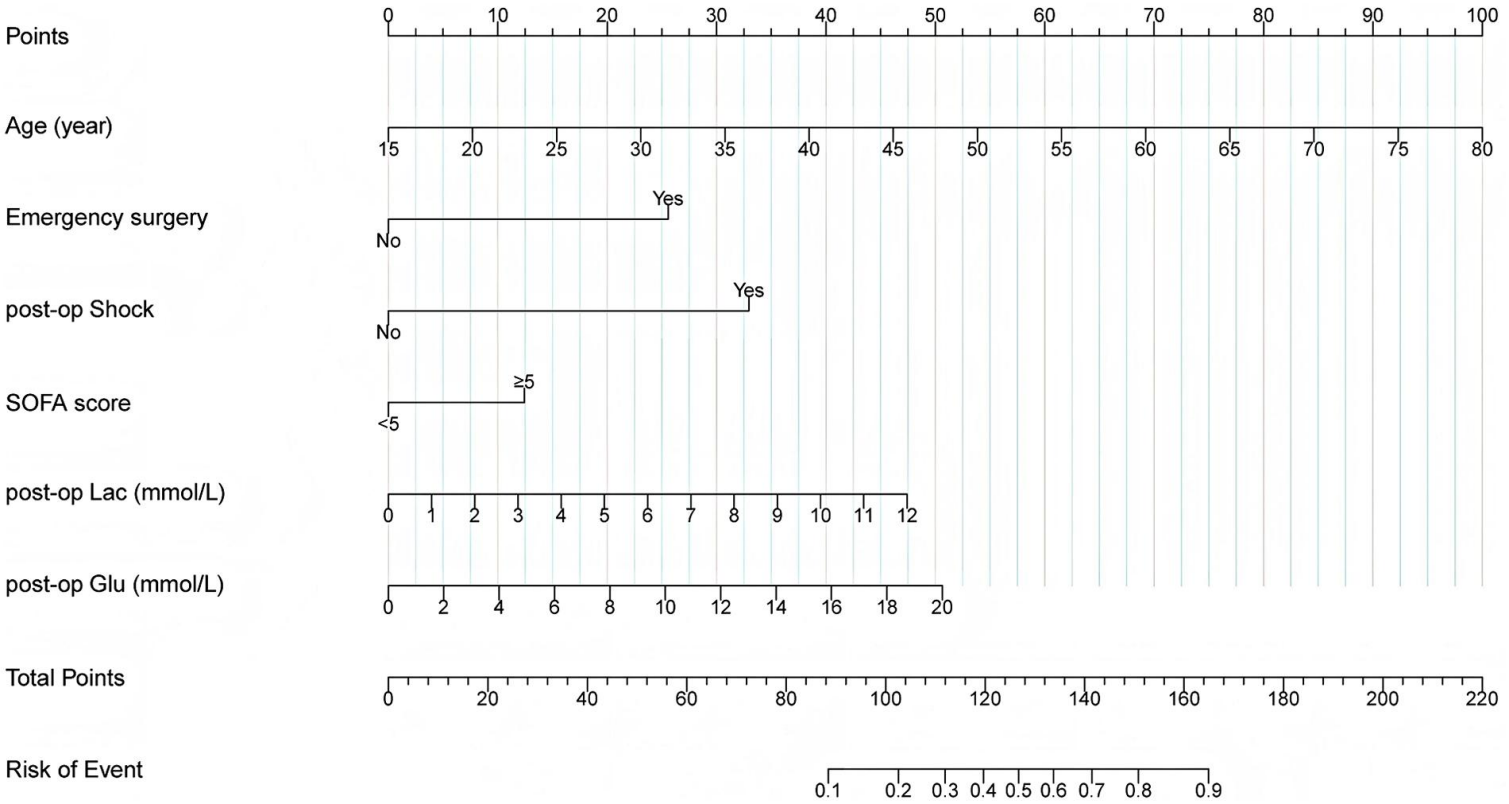
The construction of POD prediction nomogram.

**Figure 4:**
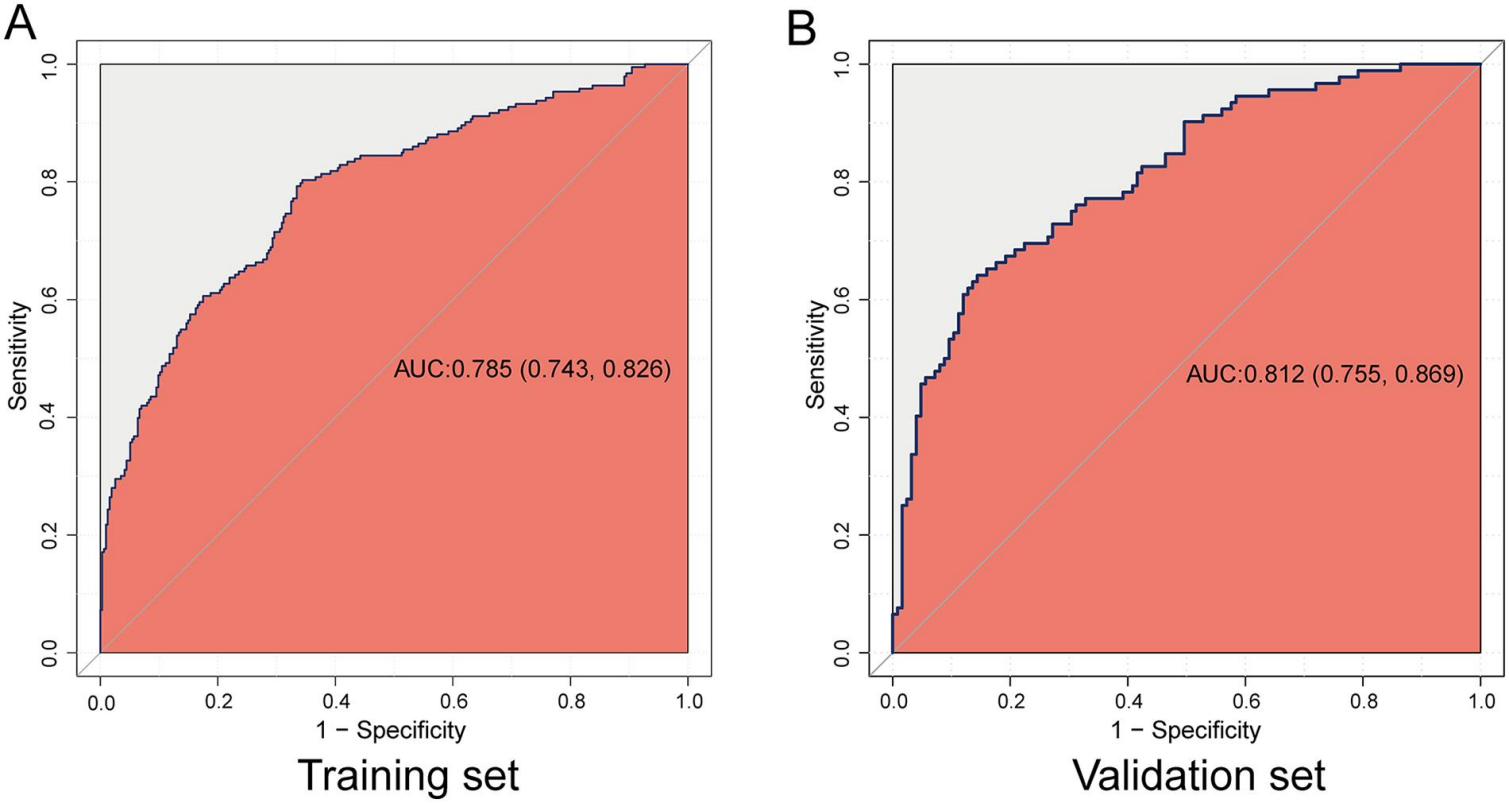
Discriminative performance of the prediction model assessed by area under the receiver operating characteristic (AUROC). **(A)** In the training set, the model yielded an AUC of 0.785 (95% CI: 0.743–0.826). **(B)** In the validation set, the model achieved an AUC of 0.812 (95% CI: 0.755–0.869).

**Figure 5:**
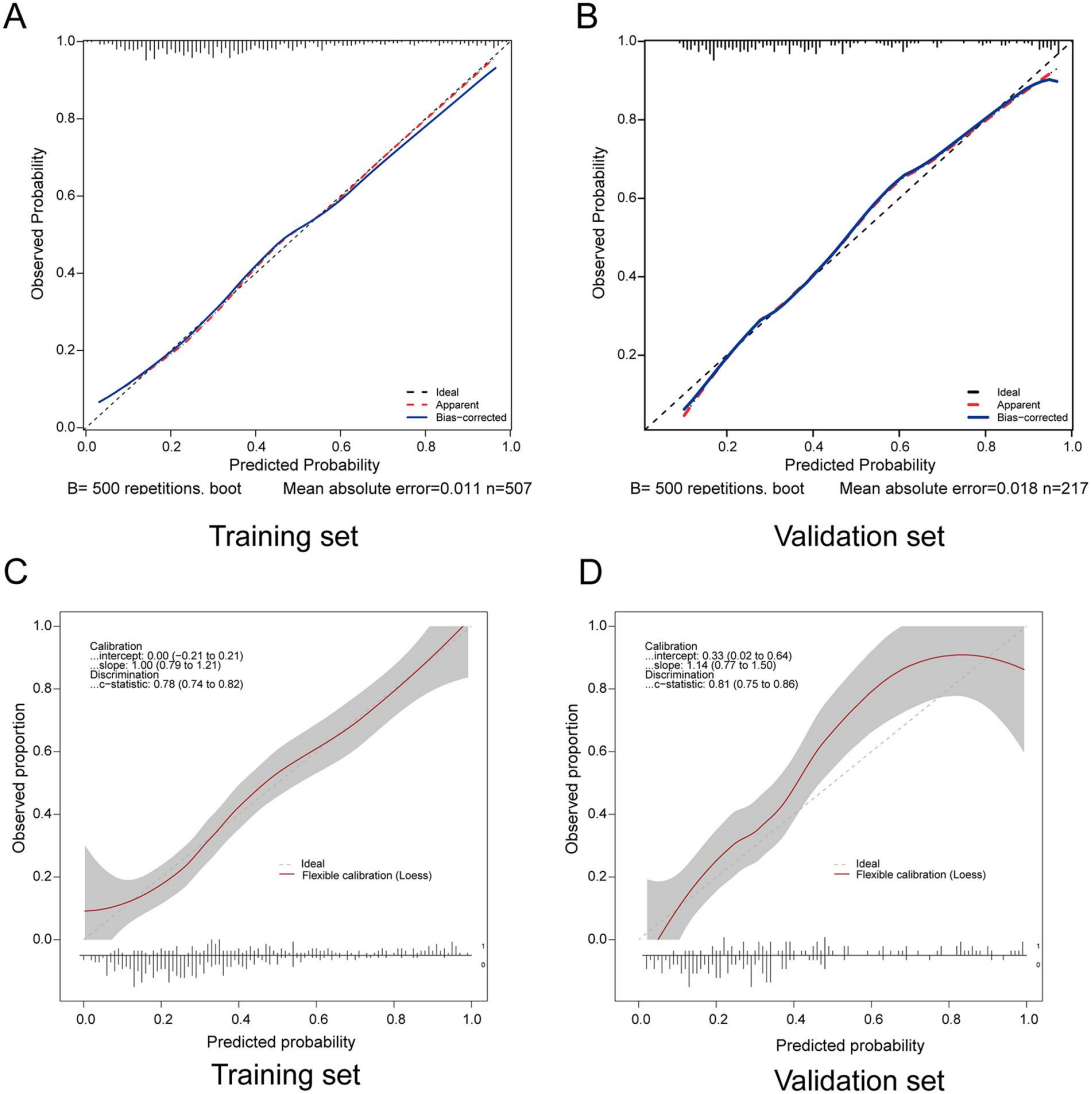
Calibration assessment of the prediction model in the training and validation sets. **(A,B)** Bootstrap calibration curves for the respective datasets. The ideal line is shown as a black dashed line, the apparent line as a red dashed line, and the bias-corrected line after 500 bootstrap resamples as a blue solid line. **(C,D)** Flexible calibration curves for the training and validation sets. The fitted calibration curve is shown by the red solid line, the ideal reference line by the gray dashed line, and the shaded area indicates the 95% confidence interval.

**Figure 6:**
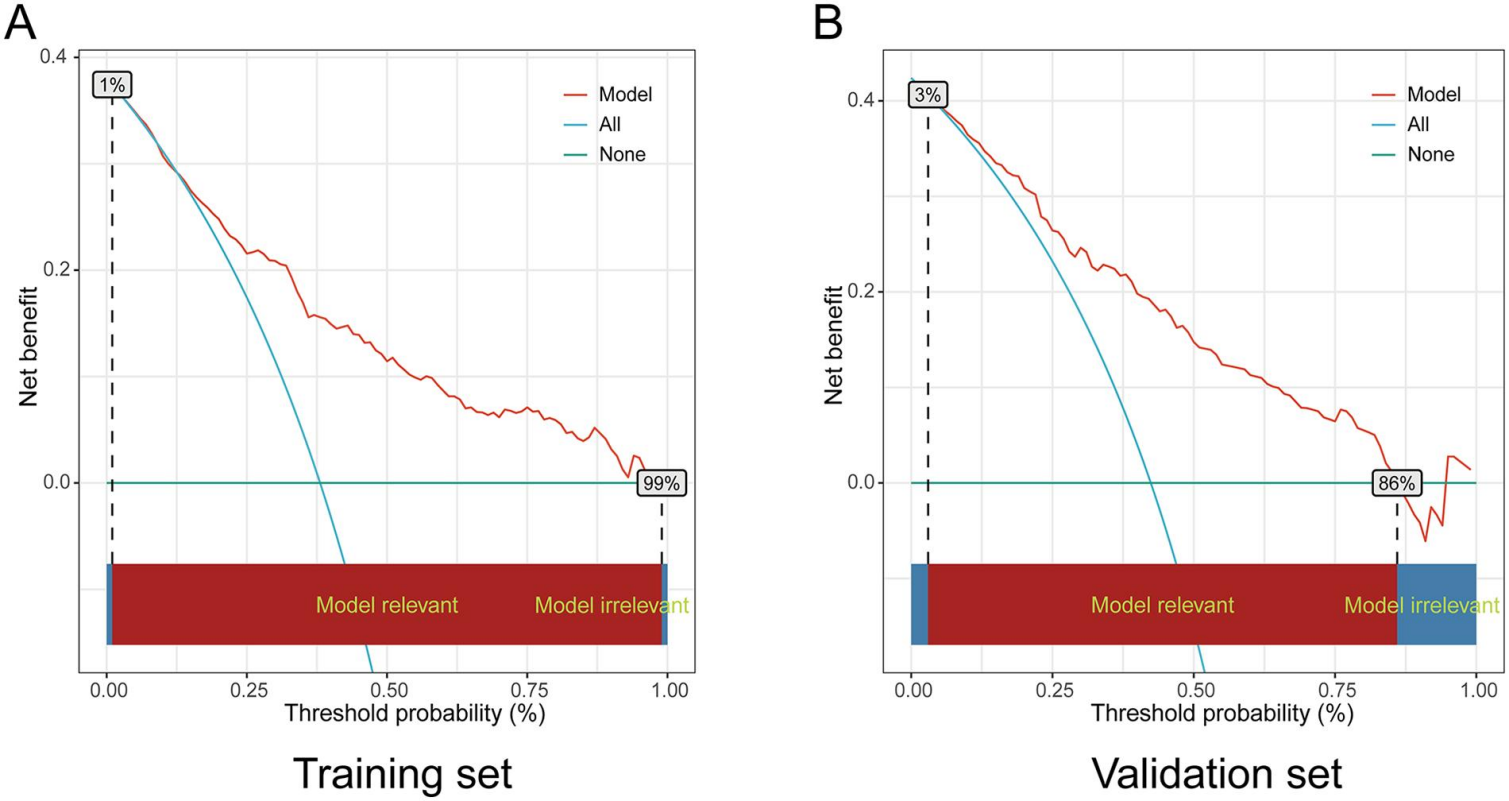
The decision curves analysis of the nomogram in the training **(A)** and validation sets **(B)** the red line represents the net benefit of the nomogram, whereas the blue and green lines represent the strategies of treating all patients and treating no patients, respectively. In both the training and validation sets, the model showed clinical utility across the corresponding ranges of threshold probabilities.

**Figure 7:**
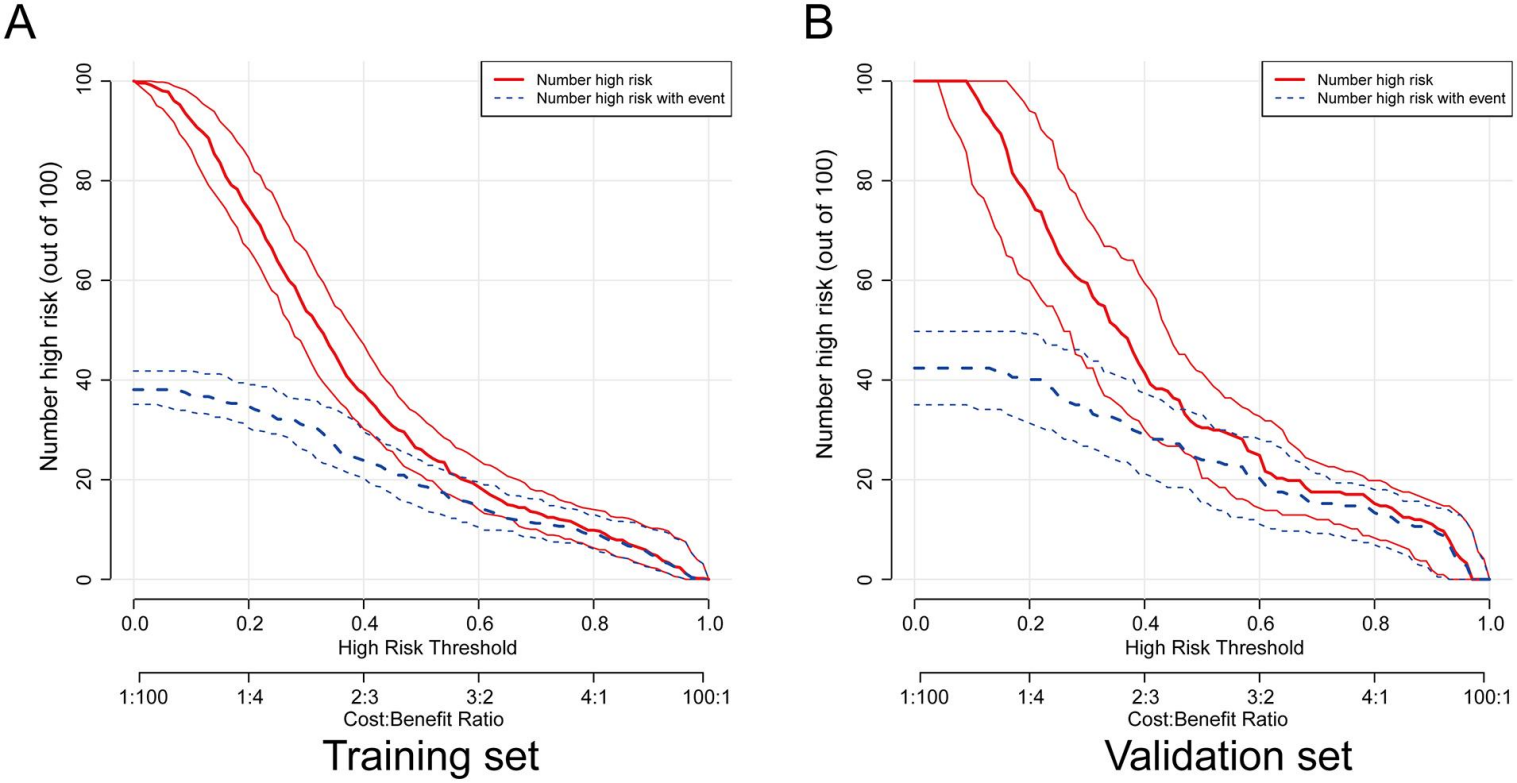
The clinical impact curve of the nomogram in the training **(A)** and validation sets **(B)** the red solid line denotes the number of subjects considered high risk at each threshold probability, and the blue dashed line denotes the number of true-positive cases within the high-risk population.

To estimate the risk of POD, a predictive nomogram was constructed using the six identified independent predictors: emergency surgery, post-op Shock, age, SOFA score, post-op Lac, and post-op Glu (Figure 3). In this model, each predictor was assigned a specific score, and the cumulative total corresponded to the predicted probability of developing POD. The full regression equation with coefficients was as follows: logit (Probability of Postoperative Delirium) = −8.607 + 1.469 ×  Emergency+1.893 ×  post-op Shock+0.088 ×  Age+0.715  ×   (SOFA≥5) + 0.227 ×  post-op Lac+0.145 ×  post-op Glu. The nomogram demonstrated strong predictive ability, with an AUROC of 0.785 (95% CI: 0.743–0.826) in the training set and 0.812 (95% CI: 0.755–0.869) in the validation set (Figure 4). The bootstrap-derived calibration results showed very small mean absolute errors in two sets, indicating that the nomogram has minimal predictive error and highly reliable probability estimates (Figures 5A, B). In the training and validation datasets, the C-index values were 0.78(95% CI: 0.74–0.82) and 0.81(95% CI: 0.75–0.86), respectively, revealing that the nomogram demonstrated strong discriminative ability to identify high-risk individuals in both datasets (Figures 5C, D). Overall, excellent consistency between predicted probabilities and observed results was shown by the calibration curves in both cohorts. Furthermore, DCA indicated a net benefit over “treat-all” and “treat-none” strategies at threshold probabilities of 0.4–93.5% in the training set and 1.9–86.3% in the validation set, confirming the clinical utility of the model (Figure 6). The CIC revealed convergence between predicted and true high-risk cases at higher thresholds, indicating good intervention efficiency and supporting the strong clinical utility and generalizability of the nomogram (Figure 7).

## 4 Discussion

POD is a serious neuropsychiatric complication with a multifactorial etiology and no single dominant pathogenic pathway (23, 24). Currently, fully effective treatments for POD remain elusive, necessitating the development of robust tools for early risk stratification. Unlike previous studies that predominantly relied on preoperative indicators, the present study integrated a comprehensive set of 23 variables spanning the preoperative, intraoperative, and postoperative phases. Six independent predictors of POD were identified by LASSO and multivariate logistic regression analyses, including age, SOFA score, emergency surgery, post-op shock within 24 h of surgery, and 24-h postoperative levels of glucose and lactate. By incorporating predictors from multiple perioperative stages, our model offers a more holistic screening approach than traditional models, providing valuable guidance for targeted clinical interventions. Although this was a single-center retrospective study, we performed internal validation to confirm the stability and predictive performance of the model. We acknowledge that differences across institutions in ICU management, sedation strategies, and delirium prevention practices may influence the incidence of delirium and, consequently, model performance. However, in our center, ICU management, sedation strategies, and delirium prevention measures follow standardized protocols. And delirium assessments are routinely performed at regular intervals according to standard practice. Therefore, despite being derived from a single center, the model remains clinically useful. In recent years, enhanced recovery after surgery (ERAS) guidelines for cardiac surgery have emphasized that early identification and prevention of postoperative delirium are critical components of enhanced recovery pathways (25, 26). Our model provides an objective early postoperative risk assessment for critically ill cardiac surgical patients and may complement ERAS-related recommendations.

Age is an established independent predictor of delirium (13). Previous studies suggest that advancing age alters blood-brain barrier permeability, allowing inflammatory mediators triggered by peripheral surgery to more readily enter the brain (27). Additionally, brain aging is associated with dysregulation of cholinergic neurotransmission (28). The combined effects of these processes predispose older patients to delirium. Consistent with this evidence, the prediction model developed in the present study confirmed that advanced age is correlated with a higher risk of POD development following cardiac surgery. Therefore, in cardiac surgical procedures involving older patients, careful preoperative assessment is essential, along with intraoperative strategies to minimize the use of delirium-inducing medications and enhanced monitoring during postoperative management.

Alterations in glucose metabolism serve a pivotal regulatory role in the pathogenesis of delirium, particularly during its early stages (29). The brain is highly sensitive to fluctuations in blood glucose levels (30). Elevated blood glucose levels disrupt the intracellular redox balance in neurons, promoting the generation of free radicals while impairing their clearance. This oxidative stress contributes to neurotoxicity and ultimately compromises neural function (31). Previous research has identified hyperglycemia as a significant factor for POD (32). In accordance with these findings, this study also confirmed that elevated post-op Glu levels can independently predict delirium following cardiac surgery, with greater deviations from the normal glucose range correlating with a higher risk of POD. The results highlight the importance of maintaining strict glycemic control and ensuring nutritional balance throughout the perioperative period.

Lactate level is a key indicator of tissue perfusion and a significant predictor of postoperative complications (33, 34). Existing reports have shown that patients with elevated blood lactate levels have a markedly increased probability of incurring postoperative complications (35). Lactate levels are also associated with the duration of ICU stay and the occurrence of major adverse events in surgical patients (36). According to previous studies, interventions aimed at reducing elevated preoperative and intraoperative lactate levels may decrease the occurrence of postoperative complications (37). Our findings further support and extend previous observations that postoperative blood lactate level is an independent predictor of POD. These results highlight the clinical significance of continuous lactate monitoring across the entire perioperative continuum as a potential strategy to mitigate delirium risk.

Emergency surgery is widely recognized as a significant risk factor for POD. Published studies indicate that patients undergoing emergency surgery have a 20%–45% probability of developing POD, representing approximately a 1.5-fold higher risk compared to that in patients undergoing non-emergency surgeries (38). This risk is even greater among older adults, in whom the incidence of POD after emergency surgery substantially exceeds that after elective surgery (39). These observations are in line with our results, where emergency surgery was significantly related to an increased incidence of POD. These further highlights the importance of rigorous perioperative monitoring and targeted preventive measures in this high-risk group.

Hemodynamic instability substantially affects neurological function. Shock initiates a cascade of oxidative stress and neuroinflammation that contributes to neuronal injury (40, 41). This pathophysiological relationship is reflected in the SOFA score, where higher values indicate multi-organ dysfunction, including neurological impairment (42). In the present study, stratification using a SOFA threshold of 5 showed that scores ≥ 5 were associated with a significantly increased risk of POD. These findings reinforce the need for intense clinical vigilance and proactive management in patients presenting with shock or elevated SOFA scores.

Several limitations of this study should be acknowledged. First, patients were recruited from a single center, which restricts the external validation of the prediction model and may introduce selection bias. Second, as a retrospective study, some cases with missing data were excluded. In addition, several potentially important predictors, including frailty, intraoperative hypotension, transfusion burden, anesthetic depth, cerebral oxygenation, and the Bispectral Index (BIS), were not systematically collected, which may restrict the generalizability of these results. Third, although four of the six predictors retained in our final model were postoperative variables, this does not imply that preoperative or intraoperative risk factors are less informative for delirium prediction. At the same time, we also recognize that, compared with intraoperative and postoperative variables, preoperative variables are more amenable to optimization and intervention. In future work, we will consider developing stage-specific delirium prediction models based solely on preoperative or intraoperative variables to enable earlier risk stratification at different perioperative time points. Therefore, the results should be interpreted with caution until confirmation in prospective, multicenter studies.

In summary, this study identified six key variables, including emergency surgery, post-op Shock, age, SOFA, post-op Lac, and post-op Glu, as independent predictors of POD following cardiac surgery. The nomogram constructed from these factors showed favorable discriminative ability and calibration. The results indicate that the model offers effective and clinically meaningful support for the early detection of high-risk individuals, thereby allowing prompt interventions aimed at reducing POD incidence. For patients identified with high POD risk, early multimodal preventive interventions may be implemented. These include optimizing sedation strategies, strengthening glycemic control, initiating early mobilization, and avoiding deliriogenic medications. Embedding the prediction model into the electronic medical record system could provide real-time risk alerts. For older patients undergoing emergency cardiac surgery, closer postoperative monitoring of glucose, lactate, and blood pressure may be beneficial. Maintaining stable glucose levels and adequate perfusion may reduce the incidence of postoperative delirium.

## 5 Conclusion

We developed a nomogram to predict the incidence of POD in patients undergoing cardiac surgery. This model provides individualized risk assessment, enabling the early identification of high-risk patients prior to delirium onset. By facilitating early clinical intervention, the nomogram has the potential to reduce the length of hospital stay and decrease the incidence of complications, thereby improving overall clinical outcomes.

## Data Availability

The datasets presented in this article are not readily available because the data will be shared through the Chinese Clinical Trial Registry (ChiCTR) within 3 months after publication of the study results. Requests to access the datasets should be directed to through the Chinese Clinical Trial Registry (ChiCTR) within 3 months after publication of the study results.
